# Evaluation of water treadmill training, lunging and treadmill training in the rehabilitation of horses with back pain

**DOI:** 10.1186/s12917-025-04950-2

**Published:** 2025-07-29

**Authors:** Tobias Geiger, Liesa Lindenhahn, Julien Delarocque, Florian Geburek

**Affiliations:** 1 Clinic for Horses, University of Veterinary Medicine Hanover, Bünteweg 9, Hannover, 30559 Germany; 2 Tieraerztliche Klinik Für Pferde Wolfesing, Wolfesing, Germany

**Keywords:** Equine, Back pain, Rehabilitation, Treadmill training, Lunging, Pressure algometry

## Abstract

**Background:**

Data about efficacy of different training modalities during rehabilitation of horses with back pain is scarce. The aim of this study was to analyze the effect of water treadmill training, lunging and dry treadmill training in horses with back pain.

**Materials and methods:**

Eighteen warmblood riding horses referred with confirmed clinical signs of back pain including abnormal responses to passive mobilisation were randomized into three groups to participate in a 6-week water treadmill training, lunging or dry treadmill program under otherwise identical conditions. Two clinicians, one blinded to the program, performed a structured clinical examination of the back at three time-points (baseline, week 3, week 6). Mechanical nociceptive thresholds were determined with pressure algometry.

**Results:**

Visual muscle development scores for the lumbar region (*p* = 0.001) and palpation sensitivity scores along the thoracic and lumbar region significantly improved at week 6, compared to baseline (*p* < 0.001). No differences in mechanical nociceptive thresholds were detected between water treadmill training, lunging and dry treadmill training at any time-point (*p* > 0.05). At week 3 and 6 of all programs mechanical nociceptive thresholds significantly increased at the level of the 10th to 18th thoracic (T18) and 3rd lumbar vertebra (L3) compared to baseline.

**Discussion/Main limitations:**

Small group size, lack of control group with ridden rehabilitation exercise.

**Conclusions:**

Different training programs without a rider could be beneficial for horses with back pain. Water treadmill training seems equivalent to dry treadmill training and lunging to increase mechanical nociceptive thresholds in the region with main saddle contact.

**Supplementary Information:**

The online version contains supplementary material available at 10.1186/s12917-025-04950-2.

## Introduction

Back pain is a common symptom in low-performing equine athletes [1, 2]. This prevalent and multifactorial condition is linked to chronic pain, impaired performance, behavioural changes, and nonspecific lameness—observed as subtle hindlimb lameness in 82% of horses with back pain, according to a survey of equine practitioners [3, 4]. Rehabilitation plays a valuable role in the treatment of horses with primary back pain [5]. In humans, it has been shown that lower back pain can lead to muscle atrophy and impaired muscle activation, resulting in altered spinal mechanics. This may worsen the pain-spasm-pain cycle, contributing to greater dysfunction, reduced muscle endurance, and a longer recovery time before returning to athletic function. Rehabilitation enhances spinal stability and function while reducing pain, and is well established in human patients [6, 7].

Rehabilitation programs for equine sport horses encompass exercises designed to both alleviate pain and discomfort and promote optimal regeneration [8]. Exercise methods that have been investigated so far include dynamic mobilization exercises, lunging (LT) (with and without training aids or elastic resistance bands), hand-walking or hand-trotting (with and without ground poles) [9–14], and treadmill training on both dry (TT) and water treadmills (WT) [8, 15–22].

Hand-walking, LT, and hand-trotting are commonly recommended for early, non-ridden rehabilitation of equine back pain, depending on the horse’s fitness and training history. Lunging produces multidirectional forces and asymmetrical muscle activity [9, 23], which may initially fatigue weak tissue, so sessions should start short and progress gradually. Hand-walking may be better tolerated than repetitive circling during LT, but introducing trot in-hand requires a calm horse and a fit handler [24]. Trotting horses over ground poles enhances the range of motion (ROM) of limb joints [25–27] and increases electromyographic activity in rectus abdominis muscles [12]. Consequently, radial ground poles may effectively contribute to increasing the limb ROM during lunging [28] and potentially promote spinal stabilisation by training of the epaxial muscles [29].

Controlled exercise, weight management, limb protraction-retraction, exercises on firm surfaces and straight-line exercises are the main components of rehabilitation of equine back pain [8]. TT enhances the ROM and symmetry patterns of lumbar vertebral angles and lumbar musculature during locomotion, and therefore, might be even more beneficial than LT [30]. However, direct comparison of these two methods have been so far not investigated.

More to that, WT has been shown to be superior to TT in the rehabilitation of the back and hindlimb musculature [18, 19, 31]. Studies in humans, horses, and dogs have demonstrated that water height significantly influences kinematic responses during WT [32–39]. Water to the level of the tarsus have been shown to promote ROM in the hindlegs and flexion of the lower back while the head and neck can still be lowered to flex the cranial thoracal spine. Deeper water causes horses to raise their head, increasing cervical and cranial thoracic extension. WT (with higher water level) has been demonstrated to be beneficial in managing joint, tendon and back pain diseases in horses [8, 19, 21, 22, 39]. King et al. showed in their study that WT significantly improves the horses’ postural stability [40]. Postural stability in human patients has been effective in enhancing joint mobility, restoring normal movement patterns, optimizing muscle function, reducing secondary muscle loss, and improving proprioception [41]. In comparison to land-based exercises, water-based exercises result in greater improvements in disability and quality of life in human patients with chronic lower back pain, with sustained positive effects over time [42, 43]. In horses, there is a lack of longitudinal studies on the effectiveness of different rehabilitation programs for equine back pain and no study so far, directly compared the already established training programs.

The aim of this study was to assess three different training protocols (LT, TT and WT) for warmblood sports horses with confirmed primary back pain over a period of six weeks.

## Material and methods

### Inclusion criteria

A total of 23 warmblood horses were referred by veterinarians who diagnosed primary back pain, based on pain on palpation of the dorsal spinous processes and the epaxial musculature. The inclusion criteria were defined as persistent presence of clinical signs of back pain for a minimum of 12 weeks. Horses showing lameness were only included if they showed a maximum score of 2 on an established five-point score scale for lameness described by Edinger et al. 2010 [44]. Horses were evaluated at both the walk and trot using the following score:

- Grade 1: mild, inconsistent lameness that is only intermittently visible at the trot and not detectable at every stride or under all conditions.
- Grade 2: lameness consistently visible at every stride at a trot, but not, or only barely, apparent at the walk.
- Grade 3: lameness is already evident at the walk and at every stride at a trot.
- Grade 4: lameness is clearly visible at both walk and trot in all situations, with the affected limb bearing weight for a short duration only during walk.
- Grade 5: the horse does not bear any weight on the affected limb [44].

Horses were not included in the study if they received any rehabilitation treatment for their back pain within 4 weeks prior to the study. It was required that horses had not been administered non-steroidal anti-inflammatory drugs or corticosteroids within the 4 weeks preceding their enrolment. The horses were maintained under their normal training conditions prior to the program. For this study 18 Warmblood riding horses met the inclusion criteria (12 dressage, 3 jumping, and 3 eventing). The mean age was 10.1 years (range 5—19 years), with a mean height of 165.5 cm (range 151—176 cm) and a mean weight of 557.5 kg (range 430—640 kg). All horses were examined by the same veterinarian between August 2020 and May 2022.

**Fig. 1:**
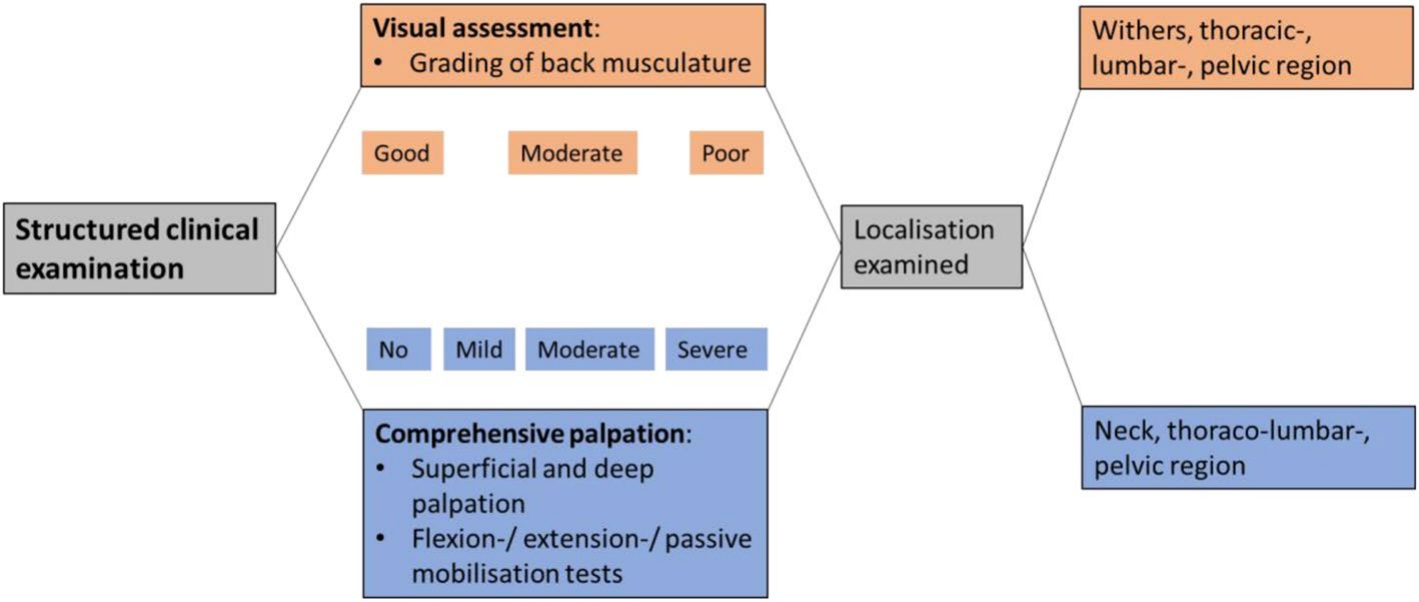
The structured clinical examination of the back involved examination of each horse at three time points. This included visual assessments (orang), and comprehensive palpation (blue)

The findings reported by the referring veterinarian were confirmed through an in-hand gait evaluation and systematic clinical examination of the back by one clinician (L.L.) (Fig. 1). This included judgement of back movements during locomotion and responses to palpation of bony and soft tissue back structures as well as responses to passive mobilisation tests. Twelve of 18 horses showed no detectable lameness, while 6 of 18 exhibited mild (grade 1/5) lameness. Of the lame horses, 3 presented with forelimb lameness and 3 with hindlimb lameness.

### Study design

The horses were randomly assigned to three different training groups—WT, TT, or LT—using a randomization list. All horses were housed in the same facility and maintained under the same conditions for 6 weeks. Their housing included boxes in an indoor stall and hourly access to an outdoor paddock. The feeding regimen consisted primarily of hay, along with the usual supplementation of grain feed and mineral supplements. The horses were not ridden for the whole study period.

**Fig. 2:**
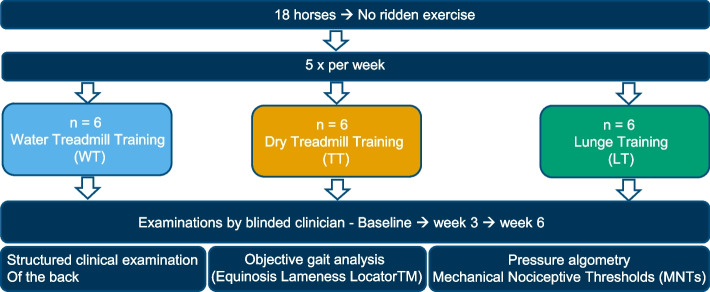
Overview of the study design

Two clinicians (T.G., L. L.), one of whom (T.G.) was blinded to the rehabilitation program and the preliminary examination, evaluated the horses using the same parameters at three different time points: (1) baseline, (2) after three weeks of training and (3) after 6 weeks of training (Fig. 2).

### Training programs

#### Lunging

All horses in this training group underwent a 30-min training session five times per week on a 20 m diameter circle on a sand and fleece mix ground (Stresan V, Stremmer Sand + Kies GmbH, Bottrop-Kirchhellen, Germany). This session consisted of a 10-min warm-up period at walk followed by 20 min of trot and canter work with 4–6 side changes and four transitions on each side. Each session followed a consistent pattern, which included a brief walk period between the trot and canter phases. Additionally, a short walk period was included at the end of each lunging session for cooling down.

The training regime included the following phases:

• Weeks 1–3: Without triangle reins and ground poles.
• Weeks 3-: With triangle reins and without ground poles.
• Weeks 5–6: With triangle reins and 4 ground poles (distance in trot with 1,3 m and in gallop with 3 m).

#### Treadmill training (WT and TT)

The treadmill used was an Aqualine Aquatrainer (ActivoMed®, Mechtersen, Germany) which was used for WT and as dry treadmill for TT. For both modalities horses were trained at a walk at five days per week for 30 min per training session. Water treadmill training started with a 5-min period without water immersion after which the treadmill was gradually filled with water to the level of the tarsometatarsal joint before proceeding with the specified speed settings (Fig. 2). The speed was set individually for each horse, depending on its reaction (e.g. head/neck position and consistent position on the treadmill) to the water, it ranged from 4.3 to 5.8 km/h (1.2–1.6 m/s) and was set individually on each day. For TT speed was also adapted individually, ranging from 4.8 to 6.4 km/h (1.3–1.8 m/s), to reach the optimal head/neck position and consistent position on the treadmill, thereby enhancing thoracolumbosacral motion [45].

### Examinations

#### Clinical examination

The horses underwent a comprehensive clinical examination following the criteria outlined by Jeffcott [46]. All examinations and evaluations were made by two clinicians (T.G., L. L.). The statistical analysis retained the scores of the more experienced and blinded clinician, excluding comparisons between the two.

This examination included visual assessment and grading of the back musculature at the level of the withers, thoracic, lumbar, and pelvic regions, along with comprehensive palpation—both superficial and deep—of the neck, thoracolumbar, and pelvic areas. Flexion, extension, and passive mobilisation tests were also part of the clinical assessment. Palpation of the cervical vertebrae was performed first, followed by evaluation of the ROM of the neck by encouraging the horse to laterally flex the head toward the ribs and to ventrally flex between the forelimbs. Bilateral compressive palpation of the epaxial musculature was performed to assess ventroflexion of the thoracolumbar spine and dorsiflexion was assessed in response to palpation of the sacroiliac region. Additional dorsiflexion of the spine was evaluated by applying upward pressure on the sternum. Continued palpation over the pelvis and the semitendinosus and semimembranosus muscles was used to elicit pelvic dorsiflexion. Unilateral palpation of the epaxial musculature was performed to evaluate contralateral lateral flexion of the vertebral column. Movement was evaluated on both hard and soft surfaces, including lunging on both reins, and assessment of back motion during stance, walk, trot, backing up, and tight turns in both directions. Signs of pain or discomfort (such as bolting, rearing, tail swishing, unruliness, rapid ear movements, or stiff, jerky motions) during back palpation, as well as a lack of improvement upon repeated digital palpation, were independently scored. Each examiner had their individual Microsoft Forms^©^ document for grading of each step of the examination. This standardized scoring form was created to record the results of the clinical examinations. Each item was scored using categorical scales or numerical ratings, depending on the parameter. The evaluators completed the form independently after each assessment, and the responses were automatically recorded in a secure Microsoft Excel database for further analysis. This digital format ensured consistency in data collection and minimized transcription errors.

#### In-hand gait examination

Visual gait assessment was conducted using the described 5 grade score for lameness [44]. Additionally, objective lameness evaluation was performed using a body-mounted inertial sensor system (Equinosis Lameness Locator™) according to the manufacturer’s guidelines and as previously described [47]. Three measurements, each with more than 25 strides on a straight and hard surface were made for each horse. Horses were excluded from the study if they showed an increase of more than 1 grade on the 5 grade score [44] between consecutive visual gait assessments and if both subjective and objective lameness evaluations indicated a significant increase in lameness during the rehabilitation period.

#### Pressure algometry

Prior to measurements, the horses were adapted to manipulations of their back. A Pain Test FDX 100 Algometer® (Wagner Instruments) with an aluminium tip featuring a 1 cm^2^ surface area was used, and results were recorded in Newton. All algometric measurements were performed by a single investigator (T.G.) to determine the nociceptive threshold. The force values obtained were reported to the clinician in a blinded fashion with the help of a second observer who noted the values displayed at the time of the horse’s discomfort response.

Each horse underwent a total of 45 measurements, taken at 15 anatomical locations along the midline and bilaterally symmetrical points over the thoracolumbar spine and pelvic region, including both exposed bony landmarks and muscular areas [48]. A fixed-order protocol of measurements was used for all horses to reduce variability between-subjects [49].

The algometer was applied perpendicular to the skin, initially in a light contact for approximately 3 s to minimize startle reactions. Subsequently, pressure was gradually increased at intervals of 2–3 s, following the protocols established by Haussler and Erb [48] ensuring smooth transitions without abrupt pressure changes.

The following reactions were considered to indicate that the Mechanical Noticeable Thresholds (MNTs) were reached: Turning of the ears, looking back at the examiner, stepping away, arching the back away from the pressure, or suddenly lifting a limb with a stomping motion or kicking. Three replicates were taken at 3—4 s intervals. If one value differed substantially from the others, e.g. due to sudden movement of the horse or slippage of the probe from the skin, an additional measurement was recorded and the aberrant value discarded. All values were noted in a Microsoft Forms^©^ document.

### Statistical analysis

Scores for sensitivity to bony back palpation, muscular/soft tissue palpation, and provocation tests were combined. Sensitivity to palpation of the dorsal spinous processes was assessed separately for the thoracic and lumbar spine, as well as the croup, with the mode of these values used for subsequent analysis. Muscle palpation sensitivity was evaluated separately for the left and right sides, and the mode for each side was calculated and used for further analysis. For the statistical evaluation of provocation tests, the mode of the thoracic and lumbar spine palpation was utilized, with the higher value selected in cases of differing scores. The same method was applied to the assessment of forelimb and hindlimb lameness.

Statistical analysis was performed with R version 4.3.1 [50]. Inter-observer reliability of scores was assessed with Cohen’s kappa coefficient with quadratic weights. The time course of ordinal scores was analysed using mixed ordinal models as implemented in the ‘ordinal’ R-package [51] with flexible thresholds and ‘horse’ as random factor. *P*-values were obtained by likelihood ratio tests.

Continuous data from pressure algometry and objective gait analysis was analysed using linear mixed model fitted with the ‘afex’ R-package [52] with ‘horse’ as random factor. Degrees of freedom were approximated with the Satterthwaite method and *P*-values obtained by F-tests with type II sums of squares. Residuals were inspected visually for departures from normality.

*P*-values were adjusted for multiple comparisons using the Holm procedure and statistical significance was accepted at adjusted *P* < 0.05. Where warranted by the omnibus test, post-hoc comparisons were performed with the ‘emmeans’ R-package [53] with Dunnett (comparison to baseline) or Tukey (all pairwise comparisons) contrasts with the corresponding adjustment for multiple comparisons. The strength of the association between time and ordinal variables and the association between pressure algometry and clinical scores in the corresponding localisations were described with Kendall’s τ_B_ [54].

## Results

### Animals

The distribution within the individual groups is listed in Table 1. One Horse was excluded after the second timepoint due to an increasing degree of lameness during the last examination.

**Table 1:** Demographics of the horses within the groups

	**WT**	**TT**	**LT**
Total number	6	6	6
Sex (S/M/G)	0/1/5	0/2/4	0/2/4
Age (y) Median	10	10,5	8,5
(Min—Max)	7—16	7—19	7—12
Median height at withers (cm)	170	165	167,5
Min – Max (cm)	159—176	163 −171	151—174
Median weight (kg)	570	542,5	555
(Min—Max)	500—630	520—610	430—640
Use (D/J/E)	5/1/0	2/2/2	5/0/1

*S* stallion,* M* mare*, G* gelding,* y* year, *cm* centimetre, *kg* kilogram,* D* Dressage, *J* Jumping,* E* Eventing, *WT* water treadmill training, *TT* land treadmill training, *LT* lunging

### Inter-observer reliability

The results of the inter-observer reliability are shown in Table 2. A substantial to almost perfect agreement was reached for all listed parameters of the clinical examination, but the muscle palpation left, which is still moderate. The analysis was performed by JD, who was not involved in conducting the clinical examinations.

**Table 2:** Interobserver reliability between 2 observers for the following clinical back examination parameters: scores for superficial muscle palpation and deep bilateral muscle palpation, visual scores for superficial musculature of the withers, thoracic, lumbar and whole superficial epaxial musculature

Parameter	Cohen’s kappa [95% CI]
Muscle palpation left	0.45 [0.13–0.78]
Muscle palpation right	0.85 [0.66–1.00]
Deep bilateral muscle palpation	0.81 [0.66–0.95]
Visual score of the withers	0.87 [0.78–0.96]
Visual score of thoracic musculature	0.75 [0.59–0.92]
Visual score of lumbar musculature	0.79 [0.57–1.00]
Whole epaxial musculature	0.86 [0.73–0.98]

*CI* confidence interval

### Development of the muscular parameters during the study period

#### Inspection of back muscles

**Fig. 3:**
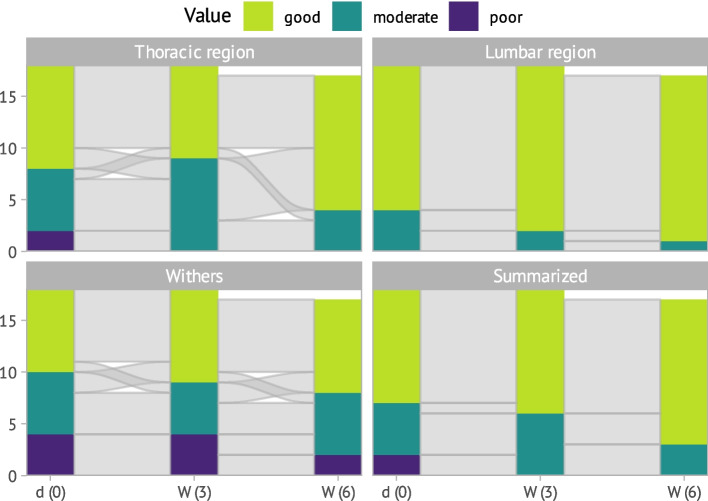
Development of visual assessment of the back muscles (at the Withers, thoracic-, lumbar region and summarized for all observed muscles) during the study period of 6 weeks shown with an alluvial plot. X-axis represents the three evaluation timepoints: d (0) = baseline, W (3) = 3 weeks, W (6) = 6 weeks. The Y-axis represents the number of horses (*n* = 18), one horse was excluded for the last examination; yellow = good; green = moderate; blue = poor

Figure 3 shows the development of scores for visual assessment of back muscles over time. These did not differ between training modalities, so that all 18 horses were assessed as one group to reach statistical significance when considering these parameters over time. Visual scores for back muscles showed significant increase in a comprehensive assessment over all regions (χ^2^(2) = 16.98; *p* = 0.001) and specifically in the lumbar region (χ^2^(2) = 15.21; adj. *p* = 0.001). Weak correlation between the increasing visual back muscle scores and time was detected (τ = 0.19).

#### Muscle palpation and muscle tone

**Fig. 4:**
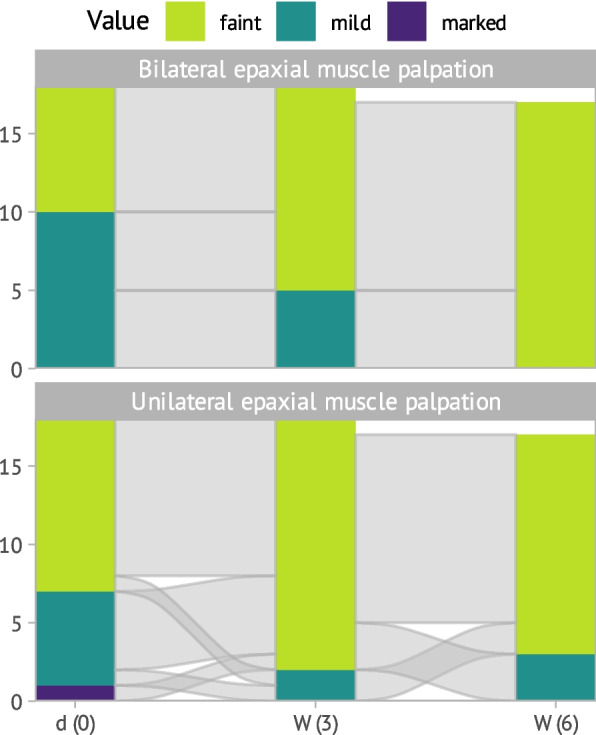
Development of back muscle palpation scores (bilateral (1) and unilateral (2)) over time during the study period of 6 weeks shown with an alluvial plot. X-axis represents the three evaluation timepoints: d (0) = baseline, W (3) = 3 weeks, W (6) = 6 weeks. The Y-axis represents the number of horses (n = 18), one horse was excluded for the last examination; yellow = faint; green = mild; blue = marked

Like before all 18 horses were assessed as one group to reach statistical significance when considering these parameters over time. A decreasing palpation sensitivity was observed over time, which did not reach statistical significance ((χ^2^(2) = 4.50; p = 0.11). This decline was observed for both unilateral epaxial muscle palpation as moderate correlation (τ = 0.20) and bilateral palpation as strong correlation (τ = 0.47), although the ordinal model did not converge (see Fig. 4). The muscle tone showed a statistically insignificant improvement over the timepoints (χ^2^(2) = 0.87; p = 0.65) and also the palpation of the musculature of the withers (τ = 0.11) and lumbar region (τ = 0.07) had a very weak correlation over the timepoints. No correlation between muscle palpation and muscle tone was observed at any level of the back spine (e.g. withers: χ^2^(2) = 4.10; *p* = 0.16).

#### Correlation between sensitivity scores for epaxial muscle palpation and the dorsal spinal processes palpation at corresponding levels

In the area of the withers (χ^2^(2) = 9.19; p = 0,03), the region of T10 – T14 (χ^2^(2) = 26.63; p = 0.00) and the tubera sacralia (χ^2^(2) = 3.76; p = 0.05) a significant correlation between sensitivity scores for epaxial muscle palpation and dorsal spinal processes was observed at corresponding levels, with a strong correlation seen in Kendal`s tau b in all three locations (τ = 0.4; τ = 0.62; τ = 0.39). For the cranial thoracic (τ = 0.40), the caudal thoracic (τ = 0.30)—although for both the ordinal model did not converge—and the lumbar region (τ = 0.45) a strong correlation between scores for epaxial muscle palpation and for dorsal spinal processes with Kendal`s tau b was found, which did not reach the statistical significance.

#### Correlation between back mobility, muscular inspection and palpation

We observed a moderate correlation (τ = 0.21) between the back mobility and muscular inspection (χ^2^(2) = 1.41; p = 0.5) and a weak correlation between the back mobility and overall muscular palpation (τ = 0.15) without converging in the ordinal model.

### Lameness evaluation over the observation time

**Fig. 5:**
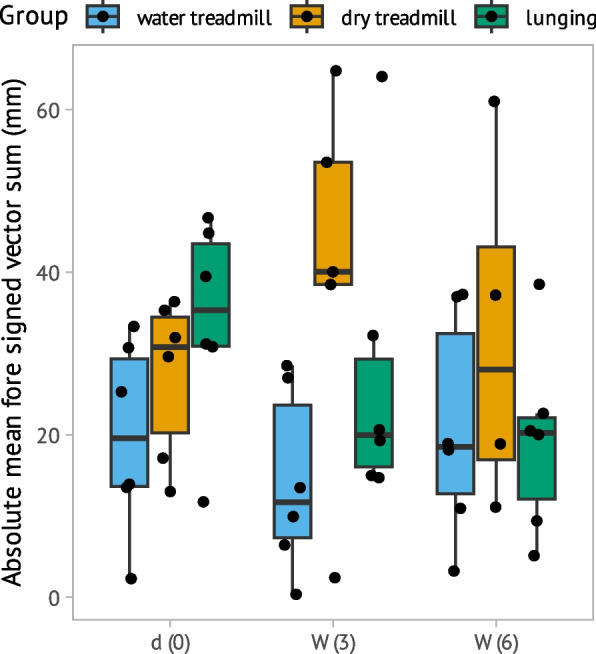
Absolute Vector sum (Y-axis: in mm) measures head movement asymmetry consistent with forelimb lameness represented with a Tukey boxplot over time (X-axis): baseline (d0); week 3 (W3); week 6 (W6). Individual data are shown as points and groups are marked with blue for water treadmill, brown for dry treadmill and green for lunging. The thick horizontal line represents the median

**Fig. 6:**
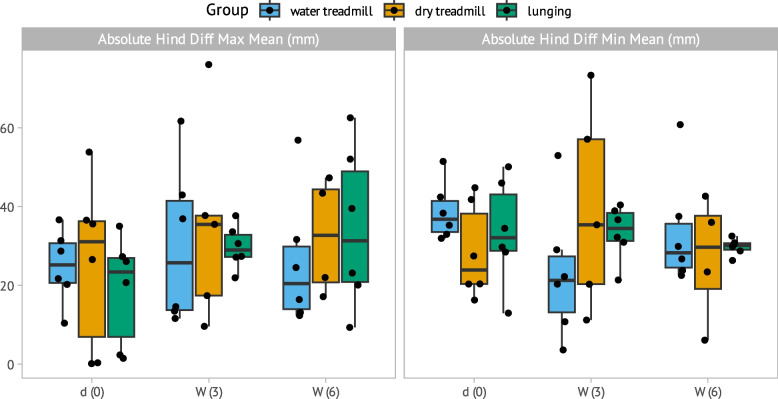
Absolute Hind. Diff. Min. and Hind. Diff. Max. measure pelvic movement asymmetry (Y-axis: in mm) consistent with hindlimb lameness represented with a Tukey boxplot over the three timepoints (X-axis): baseline (d0); week 3 (W3); week 6 (W6). Individual data are shown as points and groups are marked with blue for water treadmill, brown for dry treadmill and green for lunging. The thick horizontal line is the median

No significant difference in vector V sum (F (4.0, 26.83) = 1.40; η^2^partial = 0.17, *p* = 0.26) for forehand asymmetry was observed between baseline and any time point and between groups (Fig. 5). No significant change in absolute P Diff Max (F (4.0, 26.13) = 0.51; η^2^partial = 0.73, *p* = 0.73) and Min Mean (F (4.0, 26.32) = 1.74; η^2^partial = 0.21, *p* = 0.17) for hindlimbs was observed between baseline and any time point and between groups (Fig. 6).

### Pressure algometry

#### Pressure algometry values over the time period in the whole cohort

**Fig. 7:**
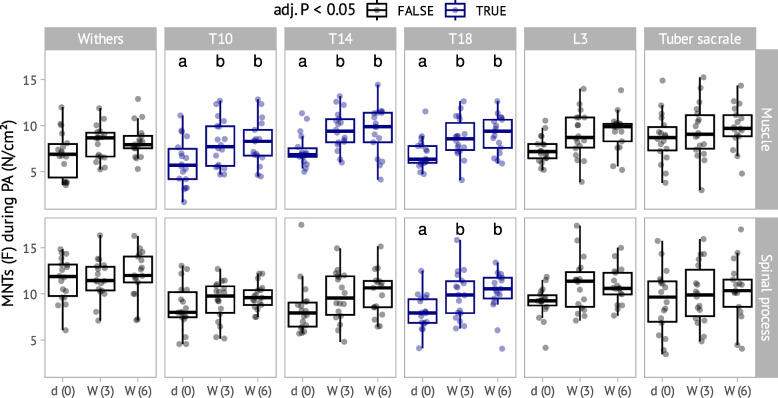
Pressure algometry according to localization (Withers, region of the 10th, 14th and 18th thoracic vertebrae, the third lumbar vertebra and the tuber sacrale) and time point. Results for the region of musculature is shown in the first and for bony surfaces in the second row. Overall there were no significant group differences between different training groups. Landmarks with significant differences over time are shown in blue (*p* < 0,05). Different letters (**a**, **b**) in each study indicate significant differences in post-hoc comparisons. X-axis represents the three evaluation timepoints: d (0) = baseline, W (3) = 3 weeks, W (6) = 6 weeks. The Y-axis represents the values of the pressure algometry in Ncm-2

**Fig. 8:**
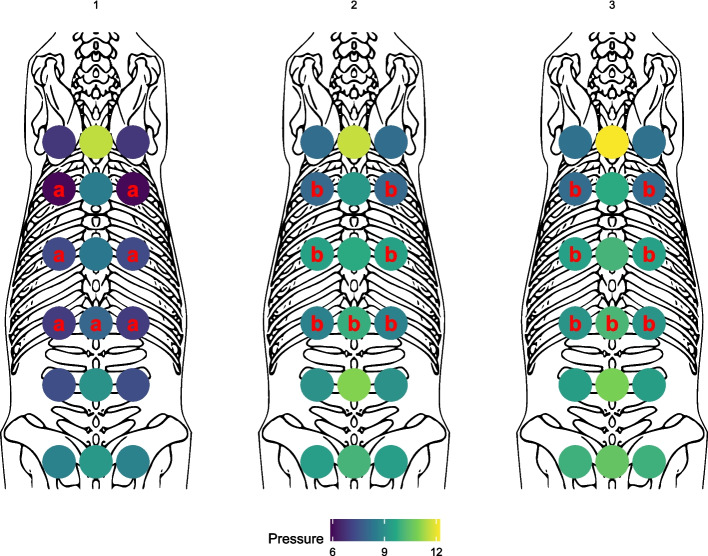
Mean difference in MNTs for all 18 horses at the different anatomical locations represented as a heatmap on an equine skeleton from above. Top represents cranial and bottom caudal. The numbers 1, 2, and 3 represent the different examination time points. Sites marked with a different red letter (**a**, **b**) differ significantly

For the pressure algometry no group differences were observed. Compared to the values obtained at baseline, higher pressure algometry values at bony and soft tissue landmarks were obtained at week 3 and 6 in the region of the caudal thoracic spine. The pressure algometry values at the soft tissue landmarks during the study period were as follows: at T10 (F (2.0, 29.10) = 16,0.67; η^2^partial = 0.52, *p* = 0.000), at T14 (F (2.0, 29.28) = 13.18; η^2^partial = 0.48, *p* = 0.001), at T18 (F (2.0, 29.02) = 8.05; η^2^partial = 0.36, *p* = 0.02) and for the bony points just at T18 (F (2.0, 29.16) = 7.21; η^2^partial = 0.33, *p* = 0.03). All results of the pressure algometry are shown in Figs. 7 and 8.

#### Correlation between the pressure algometry results and clinical findings

Mild correlations were found between the pressure algometry values and scores for muscular palpation in the following regions: T10: τ = 0.17, T14: τ = 0.18, T18: τ = 0.22. For the remaining sites Kendal’s tau b showed values below 0.1 indicating a very weak correlation. The tubera sacralia had the strongest correlation between MNTs and a pain reaction during the clinical examination (supplemental Fig. 1).

## Discussion

Our study evaluated the impact of three distinct training programs (WT, TT and LT) on a group of 18 horses with clinical signs of back pain. Specific inclusion criteria were established to ensure a consistent and targeted selection of participants, allowing for a focused investigation into back pain in horses while minimizing the influence of external factors on the results. The results indicate that all three training regimens had a significant positive effect on various clinical examination parameters. This includes an improvement of visual muscle scores for the back, a reduction in the horses’ adverse reactions to palpation of the back muscles and a reduction in parasagittal MNTs. Furthermore, a low correlation was observed between MNTs and palpation.

The results raise the question of why all three training programs produce similar clinical outcomes in horses with back pain. We hypothesize that these programs likely target specific muscle groups—such as the longissimus dorsi (LD)—which is considered a key factor in the successful rehabilitation of equine back pain. Relaxation of the LD may allow it to contract and relax appropriately, facilitating dorsoventral and lateral spinal movements in synchronization with the gait cycle [55]. The muscle mass improvement of other muscles involved in back movement biomechanics, such as musculi multifidi, iliopsoas, longus colli and scalenus muscles, ventral upper cervical muscles, and nuchal and supraspinous ligaments [56] could also contribute to clinical improvements.

According to the literature, there is evidence that all three methods have the potential to specifically strengthen the back musculature [12, 57]. Notably, the significant reduction in back pain symptoms may be associated with the substantial biomechanical impact of the LD on the entire back musculature [17, 58].

A recent study demonstrated a significant effect on the rate and size of growth of equine thoracic back profile musculature after repeated WT, correlating with increased cross-sectional areas at anatomical reference points of T5, T9, T14, and T18, three centimeters lateral to the dorsal midline [59]. These results align with our visually assessed improvement in the anatomical area where the LD is located. Therefore, future studies may try to conduct objective examinations such as measuring the cross-sectional area via ultrasound or curved ruler to assess muscle mass gain. Future studies including bigger cohorts of horses with back pain are necessary to further investigate this hypothesis.

A water level at the tarsometatarsal joint was chosen because at this height increased flexion and extension of the spine occur while horses are still able to lower the head to promote cranial thoracic flexion [39]. It has been demonstrated that increased water depth enhances thoracolumbar flexion [39]. However, other researchers have recommended limiting water depth to the level of the tarsal joint in horses recovering from impinging dorsal spinous processes [21]. The benefits of straight-line exercises, such as TT for rehabilitating horses with back pain have been well documented by other research groups [8]. Despite this, lunging remains the most used training method, primarily due to its low cost and minimal time requirements. For this reason, we included lunging as a comparator in this study, even though previous research suggests that during recovery from back pain or spinal surgery. horses should first complete at least one month of straight-line exercise [8]. Significant spinal instability was considered unlikely in our population due to the inclusion criterion of a chronic (> 3 months) history of back pain. However, in horses with limited thoracolumbar flexion–extension or compromised spinal stability, straight-line work may actually be less comfortable than circular movement, such as lunging [17].

Our rehabilitation program also incorporated ground poles into the lunging sessions. Working over poles—particularly with raised poles—has been suggested to mobilize the spine [28] and recent findings indicate increased activity of the musculi multifidi in horses trotting over ground poles [60]. Interestingly it has been shown walking over ground poles increases electromyographic activity in rectus abdominis muscles and in longissimus dorsi muscles while trotting mainly increased activity in rectus abdominis muscles [12], so that walking exercise over ground poles should have been considered as alternative modality. Although the current study design included walking during both WT and TT sessions, LT was performed at a trot and canter to mimic a basic rehabilitation program commonly implemented by owners in practice. Additionally, circular locomotion presents significant biomechanical challenges to the horse, and there is currently no evidence on how the use of poles interacts with this type of movement. Therefore, lunging over poles requires a skilled handler to prevent uncontrolled behaviour that could compromise the intended therapeutic benefits or result in injury. However, there is limited evidence in the literature supporting the specific benefits of lunging in the rehabilitation of horses with back pain, making it difficult to distinguish its effects from those of general unridden exercise.

The current study has revealed a substantial improvement of sensitivity to pressure algometry across all three training modalities in the T10-T18 region. Higher values in the pressure algometry can be interpreted because of an improvement of back pain or as an increase of muscle mass in the tested area, although we do not have other objective measured values, like muscle-thickness or pain score.

The thoracic region, including the withers, experiences the highest peak pressures due to saddle contact [61, 62]. As the horses did not carry a saddle and were not ridden during the study period, the absence of these factors might have contributed to increased thoracic spine MNTs. For example, a study comparing rider positions during rising trot revealed that the ROM of spine angles decreased significantly in the T12-T16-L2 region during the sitting phase and increased in T6-T12 and L2-L5 areas [63]. Additionally, increased pressure from the rider seems to restrict back movements under the saddle. This suggests that horses may adjust their back movements to counteract forces induced by the acceleration of the rider [63]. While a healthy equine back can compensate for these forces, horses with back pain, displaying different movement patterns [64], might experience adverse effects.

Based on previous findings, the rider exerts the greatest influence on the region between T12 and L5 [63]. Our findings align with this, as we observed low MNTs in the significant regions of T10, T14, and T18 prior to the training period, with significantly increased values measured after the 6-week training period. This supports the hypothesis that horses with back pain could benefit from rehabilitation programs that exclude saddles and additional weight. However, we did not systematically assess saddle fit or conduct analyses of the rider-horse interaction. Nevertheless, these factors remain critical in maintaining equine back rehabilitation and warrant further investigation.

The challenges of interpreting individual pressure algometry results adequately has been highlighted previously [48]. Single measurements potentially vary, so that considering trends of multiple measurements over time has been recommended. This was followed in the current study, as we always performed multiple measurements at each location at each examination date and demonstrated consistent improvements in pressure algometry values over time.

The decision to use scored clinical parameters as subjective methods and pressure algometry as an objective diagnostic modality in this study was based on their clinical relevance, ease of use, and positive validation as reliable and objective methods in previous studies [65]. Pain assessment generally falls into categories of subjective versus objective and direct versus indirect methods [65]. Moreover, the variability in serial musculoskeletal pain assessment, even within the same horses or examiner, influenced this choice [66]. An indirect method, such as a facial pain scale [67] or an ethogram [66] could have been included in the study protocol as an additional subjective measure.

Horses included in the current study were diagnosed with primary back pain by referring veterinarians, being a typical scenario for people entrusted with rehabilitation measures. Diagnostic modalities included clinical examination, radiography and response to local or systemic treatment. As diagnostic analgesia was not performed and back pain was only confirmed by a comprehensive back examination it cannot be excluded that secondary back pain might have been of relevance in some cases.

Ensuring that back pain is the primary issue is particularly important for horses undergoing treadmill training. Subtle hindlimb lameness has been shown to cause slight but measurable changes in thoracolumbar kinematics in horses with experimentally induced lameness working on a treadmill. Notably, induced lameness leads to thoracolumbar hyperextension and an increased ROM in this region. Simultaneously, it reduces the ROM in the lumbosacral segment and alters pelvic rotational movement. As a result, both the degree of lameness and secondary back pain may worsen when a horse with primary limb lameness is subjected to treadmill training [68]. These findings highlight the importance of conducting lameness evaluations both prior to and during rehabilitation. An increasing lameness grade can significantly disrupt thoracolumbar mechanics and compromise rehabilitation outcomes in horses with back pain. Based on these considerations, we excluded one horse from the program. However, a systematic comparison between the side of muscular tension or spasm identified during palpation and the side of lameness in the six horses showing signs of lameness at the final evaluation was not performed. Muscle spasms on the right or left side of the back may reflect compensatory mechanisms related to ipsilateral forelimb or hindlimb lameness. Such an analysis could have provided valuable insight into the relationship between back pain and gait asymmetries. Future studies should aim to assess this correlation more systematically.

Markerless optical motion systems have been introduced to perform kinematic analyses of gait symmetry in horses [69, 70]. These systems may also be beneficial for assessing back mobility from a lateral or even dorsal perspective [70] and could have been useful for evaluating improvements in back mobility in our study population. Similarly, the absence of objective kinematic analysis for evaluating spinal ROM represents a limitation. While clinical assessment was standardized, the detection of subtle changes in thoracolumbosacral mobility is challenging due to the minimal ROM in this region. Furthermore, spinal mobility is strongly influenced by individual intrinsic factors of each horse, making it difficult to detect condition-related changes without precise motion analysis tools [71, 72]. Incorporating such systems in future studies could improve the evaluation of cervical and thoracolumbar mobility and provide a more objective measure of treatment effects.

In this study, several general limitations are worth noting. First, the small number of horses and individual variations made it difficult to detect statistical differences between the three groups. This limitation was partly due to relatively strict inclusion criteria, being an essential prerequisite for comparison of the different training programs, however impeding horse recruitment. Second, the absence of a control group of horses being subject to unrestricted exercise, i.e. pasture turnout, or to ridden exercise is a limitation that should be addressed in future studies.

Third, the relatively short training period may be criticized. However, we aimed to offer a realistic timeframe for a rehabilitation phase in otherwise healthy horses. In the current equestrian sport, where event seasons are short, efficient rehabilitation is highly valued by trainers and horse owners. In this context, training sessions at a trot could have been included during WT and TT. Nevertheless, it is worth exploring how the same study participants develop in terms of clinical problems after returning to daily use to support our hypotheses.

Fourth, radiographic imaging was not performed systematically prior to inclusion, which may have helped identify underlying osseous pathology contributing to back pain and potentially influenced treatment outcomes.

Only one examiner was blinded to the rehabilitation program as the other examiner was entrusted with coordination of the study and consistent training of the horses. Finally, without conducting a kinematic analysis of back movement or objective measurements of the epaxial musculature, it could not be differentiated in greater detail among these three rehabilitation modalities.

## Conclusion

In summary, all three training methods led to improvement of clinical and pressure algometry values in horses with clinical signs of primary back pain. No clear preference between the three different training methods was found. The choice may depend on factors such as cost and access to specialized equipment. Controlled exercise, continuous training, and non-ridden exercises are particularly important in providing relief to equine with primary back pain.

## Supplementary Information

Supplementary Material 1: Supplemental Figure 1. Correlation between signs of discomfort during palpation of the lumbar region and Minimum Noticeable Thresholds (MNTs) during pressure algometry (PA) (F, force).

## Data Availability

The datasets used and/or analysed during the current study are available from the corresponding author upon reasonable request.

## Abbreviations

LT: Lunging
TT: Dry treadmill training
WT: Water treadmill training
Range of motion: ROM
MNT: Mechanical noticeable threshold
LD: Longissimus dorsi muscle
