# Supplementary figures and images for: Evaluation of water treadmill training, lunging and treadmill training in the rehabilitation of horses with back pain
Source: BMC Vet Res. 2025 Jul 29;21:495. doi: 10.1186/s12917-025-04950-2 (PMC12305931; doi:10.1186/s12917-025-04950-2)

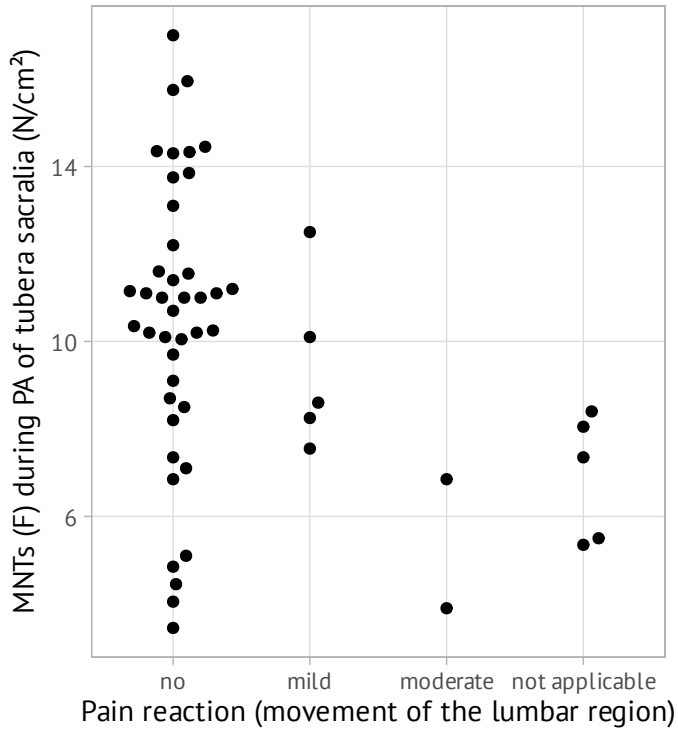

Supplement: Supplementary file 1 — Supplementary Material 1: Supplemental Figure 1. Correlation between signs of discomfort during palpation of the lumbar region and Minimum Noticeable Thresholds (MNTs) during pressure algometry (PA) (F, force). [file 12917_2025_4950_MOESM1_ESM.pdf]
